# How Older Citizens in Germany Perceive and Handle Their Food Environment—A Qualitative Exploratory Study

**DOI:** 10.3390/ijerph17196940

**Published:** 2020-09-23

**Authors:** Jana Rueter, Susanne Brandstetter, Janina Curbach, Verena Lindacher, Berit Warrelmann, Julika Loss

**Affiliations:** Medical Sociology, Department for Epidemiology and Preventive Medicine, University of Regensburg, Dr.-Gessler-Straße 17, 93051 Regensburg, Germany; susanne.brandstetter@ukr.de (S.B.); janina.curbach@ukr.de (J.C.); verena.lindacher@ukr.de (V.L.); berit.warrelmann@schule.bremen.de (B.W.); julika.loss@ukr.de (J.L.)

**Keywords:** perceived food environment, nutrition, older citizens, community

## Abstract

Apart from individual factors like knowledge or personal motivation, the environment also influences a person’s eating behaviour. Food environments can be described as the collective physical, economic, policy and sociocultural surroundings, opportunities and conditions that influence people’s food choices and nutritional status. In order to explore how older citizens in rural Germany perceive and handle their food environment, we conducted semi-structured face-to-face interviews with 35 older adults (71 ± 7 years), asking about micro-, meso- and macro-level influences on eating habits. Participants reported social factors to be crucial in shaping their diets, such as preferences of family members or social expectations connected to roles (guest, host). On a physical level, structural aspects and resources in their nearby surroundings influenced shopping and eating behaviour (for example access to an own vegetable garden, local shopping facilities and restaurants). Macro-level influences such as the food industry were hardly mentioned. Participants noticed that the environment affects their diets but dealt with undesired influences using strategies of adaptation and behaviour change, rather than challenging the environmental influences. Public health projects should raise the awareness of the multiple environmental influences on eating behaviour and also help people to create healthier food environments.

## 1. Introduction

The prevalence rates of obesity and nutrition-related non-communicable diseases (e.g., cancer, type 2 diabetes, cardiovascular diseases) are increasing in many developed countries, highlighting the necessity of public health programs focusing on healthy diets [1,2,3,4]. Educational and/or behavioural approaches, however, have not been as successful as expected [5]. This can partly be attributed to the fact that not only individual factors (e.g., knowledge, personal motivation) affect a person’s eating behaviour, but also the environment and the conditions that the person lives in. In 1986, the Ottawa Charter, published by the World Health Organization, stated that “health is created in and lived by people within the settings of their everyday life”; it suggested that health promotion approaches should aim at changing people’s surroundings and contexts in a way that renders healthy choices easier [6]. Food environments can be described as “the collective physical, economic, policy and sociocultural surroundings, opportunities and conditions that influence people’s food choices and nutritional status”. [7,8] US-researchers also described “obesogenic environments”, which facilitate high energy intake and sedentary behaviours and thus promote overweight and obesity in individuals or populations, e.g., by a high number of fast-food outlets in a neighbourhood [9,10,11]. Availability and affordability of healthy foods have indeed been shown to affect food choices, eating behaviour and/or body weight [12,13,14,15]. In the US, the term “food deserts” was coined for regions with limited access to affordable healthy food (e.g., fruit and vegetables). Studies implied an inverse correlation between the local presence of supermarkets offering a wide range of fresh produce and rates of overweight/obesity in the respective area. In contrast, prevalence of overweight and obesity is increased in areas with high numbers of convenience stores selling highly caloric products [16,17,18].

In order to describe the multiple factors influencing what people eat, and with a view to creating healthy food and eating environments, Story et al. [19] suggested an ecological framework including social (e.g., support of family and friends), physical (e.g., availability of food) and macro-level environments (e.g., the food industry or cultural traditions). A similar conceptualization was proposed by Glanz et al. [20]. It emphasizes the role of the “community nutrition environment” on the one hand, including number, type, location and accessibility of food outlets, and the “consumer nutrition environment” on the other hand, including the availability and affordability of healthy food options and has frequently been cited by other studies [21,22].

Although these multifaceted frameworks have highlighted the complexity of factors influencing food choices, the bulk of research focuses on single or selected components of the local food environment (e.g., the presence of supermarkets in a given area) and on selected aspects of eating behaviour (e.g., consumption of fruit and vegetables) [13]. These approaches may fail to depict comprehensively the “real” food environment of a person, which can be affected by various factors (e.g., public transportation, opening hours, personal preferences, etc.) In order to better understand these factors and their mutual interactions, researchers suggested examining people’s subjective, “perceived food environment”, using quantitative and qualitative research designs [23,24,25]. For instance, Jilcott et al. [26] focused on the perceptions of US-American middle-aged women and noted that their food choices were determined by factors classified as social environment, e.g., influence of co-workers and family members.

The importance of healthy nutrition in every stage of life is obvious. Nevertheless, due to the demographic change, older citizens constitute an ever-growing proportion of society, and senior citizens in industrialized countries usually have a life expectancy of 20–30 years and more after retiring. During this period of life, the risk for chronic diseases (e.g., diabetes, cancer or arthritis) is increasing, and malnutrition is a serious condition affecting almost 46% of the European population over 65 years [27]. Biologically, ageing is associated with reduced energy intake and loss of appetite. In addition, age-related visual impairment or manual dexterity can limit the ability to buy, prepare and enjoy healthy and nutritious foods [28,29,30]. Social conditions, such as living and eating alone, can also diminish dietary quality and the amount of consumed food [31,32,33]. 

To the best of our knowledge, no European or German study has studied the perceived food environment of senior citizens so far. The purpose of this study was to explore how older people in rural communities in Bavaria, Germany, perceive their food environment. It also strove to understand how senior citizens handle different environmental factors in their everyday life, i.e., how they cope with factors that they realize influence their food choice and diet in a negative way.

## 2. Materials and Methods

### 2.1. Study Design

We used a qualitative design by conducting semi-structured face-to-face interviews with senior citizens in five German communities. 

### 2.2. Recruitment of Participants and Data Collection

The sample consisted of 35 senior citizens of 65 years and over. They were recruited among the 54 participants of community-based group projects on healthy nutrition. The aim of this participatory project was to mobilize senior citizens to plan and implement nutrition interventions for themselves or the community at large; the study framework and results of the pilot study are described elsewhere [34,35]. The group meetings took place in publicly accessible rooms of the communities, e.g., in the town hall, multi-generation house or parish halls. 83% of the participants were female, 57% of participants were married or in a relationship (see Table 1). There was just one couple attending the group meetings together, and in the interview study described here, just one person of the couple was included. Almost all of them lived in an own house in the community with fitted kitchens with a usual range of appliances (refrigerator, electric stove and oven, dishwasher). Many of them even had an own vegetable garden, but we did not ask that explicitly in the sociodemographic questionnaire. Recruitment was continued until theoretical saturation was reached, i.e., no more novel themes came up in the interviews.

Data collection took place in 5 Bavarian communities (7000–20,000 inhabitants) from 2012 to 2014. The interviews were scheduled before or after the group meetings. In some cases, a separate appointment was made for the interview, at a date and time convenient for the interviewee. All 54 group members were asked to be interviewed; 19 refused to take part in the interview study. Main reasons given for the refusal were feeling uncomfortable with being interviewed or lack of time. The interview partners were informed about the aim of the study and gave their informed consent to be interviewed and recorded. Interviews were conducted by a researcher facilitating the respective community project. At the beginning of the interview, we stressed that there were no “correct and wrong” answers to the placed questions and that we were just interested in the respective opinion of the participants. For this study, only researchers who had experience in conducting interviews and were additionally qualified in qualitative methods (e.g., workshops on qualitative research methods or different types of interviews) were employed. All interviewers received one day training specifically for the research question and the population studied (older people). In addition, there was a pilot phase. All interviewers who were involved in the study performed three pilot interviews with a convenient sample of older adults. The semi-structured interview guide (Supplementary S1) was discussed and slightly adapted afterwards. Interviews lasted between 15 and 40 min and were audio-recorded. The data material was de-identified after transcription, so none of the researchers, except the interviewer, could link the answers back to the interviewed senior citizen. The analysis and presentation of data did not allow for the identification of individual participants. 

The ethics committee of the University of Regensburg was consulted and decided that there were no ethical concerns that would require further authorized approval processes.

### 2.3. Question Guide

The interviews followed a semi-structured guide. In order to tap the participants’ views on factors influencing their food choice and eating behaviour, they were asked the following:

- To describe their typical eating behaviour on a workday and on weekends (prompts: buying food, preparing food, eating together/alone, eating out) and aspects that influence it;
- To explain the relevance of their family members with regard to food choice and eating behaviour;
- To name factors they have experienced to influence their eating and food shopping habits;
- To refer to biographical events that have (recently) affected/changed food choice and diet;
- To describe their nutritional preferences and how those have developed;
- To point out actions that need to be taken to make healthy eating easier;
- To explain what they do to make healthy eating easier for themselves or for others, e.g., family members.

For every item, the interviewer started with broad open questions allowing the participant to answer what first came to mind. Usually, the conversation continued by offering standard probes to the interviewee. When participants had trouble finding an answer, the interviewer asked more specifically, e.g., about eating behaviours on typical days with focusing on different possible environmental factors. If someone mentioned factors that influenced their eating behaviour in a negative way, the interviewer also asked how participants tried to handle these factors. 

In addition, each participant was asked about his or her age, place of birth, living situation, marital status, children and (previous) employment. 

### 2.4. Data Analysis

Descriptive data concerning demographics was analysed and Interviews were transcribed verbatim with the help of Olympus DSS Player Standard Transcription Module. The transcripts were classified according to the gender of the interview partner (f = female, m = male), and his or her marital status (*p* = “living with a partner”: in relationship or married, a = “alone”: divorced, widowed, single), anonymized and continuously numbered (Interview Partner = IP 01 - IP 35, L = Line). We conducted a content analysis according to Mayring [36] using the Qualitative Data Analysis (QDA) software ATLAS.ti Version 7 (ATLAS.ti GmbH, Berlin, Germany). 

Using an inductive approach, possible environmental factors influencing the nutritional behaviour of the interview participants were identified and coded. Parallel to this first round of analysis, a coding instruction was formulated to ensure standardized coding. We coded text passages that met each of the following criteria:

1. The interviewee reported a (social, physical or macro) environment to have a (positive or negative) influence;
2. The mentioned influence is related to the topic “food and eating” (e.g., purchase of food, eating behaviour, health-related effects of food…);
3. Text passages are reflecting the individual perceptions of interviewees and are experienced by themselves.

Text passages were not coded when interviewees spoke about environmental factors influencing nutrition-related behaviours of others, e.g., pointing out that young people’s diets were affected by TV advertising. Quotations that suggested a certain social desirability were discussed with a second researcher and possibly not included in the analysis. After that, all coded environment variables were deductively assigned to the three levels (social level, physical-material level, macro-level) of the model by Story et al. [19]. Within the three levels, these codes were again inductively logically grouped into different sub-categories. Thus, after this second round of analysis a first category system was created. The individual categories were defined before starting a further analysis, checked logically and, if necessary, subsumed in case of overlaps. If necessary, a new category was also formulated. Finally, a coding framework was developed which was used to analyse the interview-material. If new categories occurred during the analytical process, the category system was adapted accordingly. The main codes and its superior categories can be found in Table 2 (see the first two columns under “Perceived environmental factors related to nutrition”).

## 3. Results

All 35 participants (f = 29, m = 6, 71.2 ± 6.9 years) were retired; the majority had children and grandchildren. Most of the participants had a driving license and could use a car for shopping. Male participants reported to have worked in business administration, as engineers, as train drivers or pastry chefs. Four women stated to have been housewives, others had worked as teachers, in skilled occupations (e.g., seamstress, gardener or businesswomen) or on their own agricultural farm. The participants used plain everyday language to describe their perspectives on eating. 

### 3.1. The Perceived Food Environment of Older Citizens

Older citizens mentioned numerous environmental factors influencing their eating behaviour. Social factors were named most often, e.g., preferences and needs of family members. On a physical level, shopping facilities in the neighbourhood were considered sufficient to satisfy most nutrition-related needs, whereas restaurants in the neighbourhood were criticised because of their “traditional” (unhealthy) cuisine or use of additives. Influences on the macro level, e.g., the food industry, were hardly mentioned. 

Table 2 shows how the participants perceive their food environment, which effects the food environment has on their food choice, eating and shopping behaviour and how participants handle the effects of their perceived food environment.

#### 3.1.1. Social Level Environments

According to the interview partners, their families had a significant impact on their eating behaviour in everyday life. Nutrition-related needs of relatives, especially spouses, were often caused by illness or certain conditions, e.g., high blood pressure or food incompatibilities such as lactose intolerance. Interviewees also reported food preferences of family members, e.g., particular dishes that they liked (e.g., pastries, meat) or disliked (e.g., salads). Adapting the food preparation to these needs and preferences was often experienced as a limitation for the own healthy eating possibilities.

*“I have a husband who has many problems with nutrition. Lactose, fructose, (…) he doesn’t like cheese, he doesn’t like fish. How can you cook healthily then?” (IP14, L74, f, p)*

This is why some older women mentioned that they had become more independent in their food choice and mealtime rhythm after their husbands had died or had left after a divorce.

*“But now, being on my own, it’s me who is important. I can prepare the food that I want, in terms of ingredients and flavour. That makes it [healthy eating] easier”. (IP9, L104, f, a)*

*“Since I left my husband, (…) I can cook whatever I want to. Before that, I used to make traditional dishes. And now, living alone, I’m cooking in a completely different way. I’m totally into healthy nutrition”. (IP19, L53, f, a)*

*“When you don’t have to cook for the whole family anymore (…) you can be more creative with your meals”. (IP26, L65, f, p)*

On the other hand, a reduction of persons living in their households also led to quantitative and qualitative changes of purchasing and cooking habits, e.g., after the children had moved out. Almost all interviewees explained that they had to get used to cooking fewer helpings. In addition, some of the older people mentioned that a change of the family composition or household size resulted in a reduction of the variety of dishes. They explained that some dishes would not be worthwhile preparing for just one or two persons, e.g., baking their own wholemeal bread.

*“[Cooking healthy food]—since it’s just the two of us… this is more difficult. In former times, when my children still lived at home, we used to make our own bread at home, or cooked burgers made from spelt and more such things with wholegrain flour”. (IP3, L41, f, p)*

Visits of family members or family reunions are linked with a change in eating behaviour, according to the interviewed older adults, especially with the preparation and consumption of traditional, fatty food. Participants (predominantly women) reported to cook more elaborate and festive dishes (e.g., roast pork, asparagus) than if they were alone.

*“My grandson visits me on weekends, and he wants to eat things that are different from what I usually eat on weekdays. He wants his roast, he wants proper meals”. (IP18, L77, f, a)*

*“I’m inviting friends over fairly often. I’m cooking for them, we’re eating together, drinking coffee. My friends know that I always have some treat in store for them”. (IP1, L125, f, p)*

Not only being the host, but also being guest was reported to come along with unhealthy dishes and oversized portions.

*“Atmosphere was so great at the party. Wine, champagne, everything you liked. And in this moment, you can’t say no. That’s just not possible”. (IP17, L59, f, p)*

*“When I meet friends for dinner in a restaurant, and I eat a rich and hearty dish, then usually I don’t feel well later, and I have trouble sleeping. That’s just not good for me”. (IP21, L118, m, p)*

Besides family and friends, medical experts (e.g., doctors, pharmacists or nutritionists) were also mentioned as socially relevant persons who can influence eating behaviour by giving nutrition advice. The interviewees appreciated this advice and did not question its correctness.

#### 3.1.2. Physical Level Environments

According to the interviewed senior citizens, structural aspects and resources in their close or nearby surroundings (backyard, neighbourhood) influenced their shopping and eating behaviour. 

Most of the interviewees, who were recruited in a rural area, reported cultivating fruits and vegetables in their own backyard. The access to food grown in an own backyard vegetable garden was perceived to make healthy nutrition easier, e.g., due to knowing the source of one’s foods and not using pesticides.

*“This year, we have tomatoes, cucumber, currant and raspberries in our garden. Look, this is all organic and pesticide-free. And if you buy it in stores, you don’t know what’s inside”. (IP20, L24, f, p)*

*“What do I do to eat healthily? I’m cultivating my own fruit and vegetables. I don’t have to buy organic food”. (IP17, L96, f, p)*

As the availability of home-grown food is seasonal, it can affect the menu, especially when particular fruits or vegetables are in season.

*“I’m using everything that we are cultivating in the garden. Currently it’s rhubarb-time, so we’re eating a lot of rhubarb: rhubarb flan, rhubarb tarte..”. (IP3, L125, f, p)*

According to the interviewees, the neighbourhood infrastructure could influence food choices and eating behaviour by the local shopping facilities. Overall, the interviewed seniors were satisfied with the number of nearby food stores and supermarkets. The accessibility of stores, however, was sometimes described to be challenging, mainly due to insufficient public transportation.

*“On the whole, shopping facilities are fine in [this] community. But for older people, [the supermarket] is too far away, and it’s not nice to walk all the way from the city centre to get there”. (IP19, L40, f, p)*

Some interviewees criticised the range, display and pricing schemes of produce and groceries in the supermarket; advertising and special offers were described to entice them to buy unhealthy food or big family portion sizes. Furthermore, poor availability of selected foods in some supermarkets (e.g., fresh fish, whole meal flour, organic foods) was also criticised by some.

*“As senior citizen, it’s very difficult to buy cheap food for your own person. You have a small household. 2 1/2 kg oranges on offer! Just bulk-packs. That’s difficult”. (IP16, L55, f, a)*

*“Temptation is always there, always. In the supermarkets. You know, you’d love to eat it, but you know that’s not good for you!” (IP19, L73, f, a)*

The cuisine of local restaurants was perceived to be influencing food choice and eating behaviour as well, especially in a negative sense, as traditional inns with plain fare predominate in their neighbourhoods. Therefore, eating out on special occasions is often associated with choosing unhealthy meals (e.g., energy-dense foods, meat, use of glutamate and other additives, etc.), poor availability of vegetable dishes and big portion sizes.

*“If you’re ordering in a restaurant, you get very big portions of food. Way too much. And I have to throw away half of it, at least”. (IP27, L90, f, a)*

*“On the other hand, when being under way during the day, some seniors do not mind eating unhealthy snacks at takeaways and bakeries.*

*“If I’m somewhere out and about during the day and I’m hungry, and there is just curried sausage with chips…then I will be eating curried sausage with chips. Although I know it’s not healthy”. (IP17, L99, f, p)*

#### 3.1.3. Macro-Level Environments

Apart from interviewees’ needs to meet the requirements imposed by social roles e.g., host-role and guest-role, which influence eating behaviour in social interaction, participants did not mention many macro-level factors, such as the food industry or agriculture policies. Few seniors describe a lack of transparency, e.g., regarding the production of processed foods, which deters them from buying special groceries.

*“At the supermarket, you get a chicken for 1.95 €. I don’t know what kind of chicken that is. For 1.95 €. Well, I don’t like to eat that”. (IP27, L146, f, a)*

Furthermore, nutrition-related reports in mass media (TV, magazines) were described to influence diets—by endorsing to choose or avoid certain food items.

*“I’m not eating meat, or less meat... Not because I am a vegetarian. It’s because I watched those TV reports about poultry keeping and other animals, and it made me sick!” (IP26, L67, f, p)*

*“In magazines or on TV, there are so many great health reports about healthy food, unhealthy food, I am always reading this. I know a lot about this, I’ interested in it and I’m trying to put this into action”. (IP8, L109, f, p)*

On the other hand, seniors sometimes feel confused by contradictory information about healthy nutrition they are getting in the different TV shows or magazines.

*“Every day you can read something new. Two days ago, there was a report about milk. Milk was supposed to be suitable only for infants and small children. So, I’m confused. Some experts are saying, that milk is healthy. This Professor said the opposite”. (IP14, L182, f, p)*

### 3.2. Handling the Food Environment

Overall, the interviewed seniors seem to be well adapted to their food environment and have developed strategies to cope with many influencing factors. 

#### 3.2.1. Handling the Social Level Environment

Besides adapting to a smaller household by changing cooking habits, participants have developed different strategies to deal with preferences and needs of family members. Most of them also reported adaptation to preferences of their spouses.

*“He [husband] has a strong influence on me, because he dislikes and rejects many things [foods]. […] And I have adjusted to his preferences; I can’t stand the discussions anymore. I used to think: I need to do this [to argue], but now I will rather spare me that stress, that’s better for my nerves”. (IP17, L74, f, p)*

One of the women explained this behaviour with the “traditional” understanding of gender roles, according to which wives were supposed to adjust to their husbands.

*“… as long as he is still alive, you have to comply with your husband in terms of cooking”. (IP27, L44, f, a)*

Other female participants described cooking separate dishes for their husbands and themselves; they accept a higher effort in food preparation in order to maintain their independent diet. 

When they eat out on special occasions, some seniors have developed strategies that helped them live up to their social roles (as guests) and at the same time allow them to control their diets. Especially women try to compensate the (anticipated) high consumption of calories by fastening or eating only little during the day of an invitation, in order to control their weight.

*“On Monday [night], we had a big invitation. So at lunch, I just had a milkshake”. (IP17, L59, f, p)*

#### 3.2.2. Handling the Physical Level Environment

In order to address potentially negative influences that restaurant menus can have on their food choice, some interview partners report to avoid eating out completely. Others try to cope with oversized portions by ordering special plates for seniors or taking food leftovers home. 

Concerning the food shopping facilities in their communities, the interviewed seniors seem to inform themselves about the supply of groceries in the different stores of the region and organise their shopping behaviour around the availability and accessibility of particular food items (fresh fish, whole meal)—even if this entails a higher effort.

*“Every store [in our community] has fresh fruit and vegetables in store. [But] the discount stores don’t sell fresh meat and sausages, so [for that] I’m going to the supermarket, or to the butchers”. (IP19, L150, f, p)*

*“... this organic whole flour meal, it really bothers me that I can’t buy it around here. I have to cycle to [next bigger city] to get it in a special shop”. (IP2, L295, f, p)*

Putting special emphasis on the availability of seasonal and local food as well as the possibility to buy single portions of fruit and vegetables, many of the older people prefer shopping in organic stores or on farmer’s markets in the vicinity (for reasons of transportation or household economy).

*“On farmer’s market, I can buy single pieces of vegetables: 5 carrots, I don’t have to take 2 kg of them. […] I am supposed not to carry too much weight”. (IP16, L123, f, a)*

Besides, some seniors referred to situations when they fail to control factors of the physical food environment, e.g., buying irresistible offers of sweets in the supermarkets, eating/drinking too much when being invited at special occasions or eating ready-made unhealthy snacks at home.

*“It’s a catastrophe with the sweets. I can’t resist. If I have a chocolate bar at home, I’ll have to eat it all in one. I can’t control myself”. (IP6, L191, f, a)*

#### 3.2.3. Handling the Macro-Level Environment

Facing more abstract factors of the macro-level environment, e.g., the food industry, marketing strategies or labelling policies, most of the seniors felt powerless.

*“From politics, we can’t await anything [concerning the food industry]. The big companies, it’s all controlled by the food industry”. (IP25, L143, f, p)*

The feeling of being deceived, e.g., by food names and labels, made it difficult for interview partners to make informed choices and thus control their food choice. Implicitly, politicians were hold accountable for that, but most participants considered their personal behaviour a possibility to put pressure on the food industry by changing their food shopping behaviour, e.g., buying more regional and seasonal products or started eating less meat.

*“I’m taking a closer look at food labels. I’m taking more time and read what’s written on the back of a product. (…) And I’m trying to buy more regional food at the farmers market, not so many things in the supermarket”. (IP3, L14, f, p)*

*“Consumers can do a lot. I mean, they can eat less meat. (…) And I’m feeling very good since I’ve been eating less meat and sausages. Although I really like to eat meat”. (IP25, L123&157, f, p)*

One of the older people even stated that she tries to be more self-sufficient by growing her own fruit and vegetables and be less dependent from the available produce in supermarkets.

## 4. Discussion

### 4.1. Main Findings

The interviews illustrate that the participating senior citizens are aware of multiple factors influencing their food choice and diet in their everyday life, some of those factors even preventing them from eating the way they would prefer (in terms of time, ingredients and amount) or the way they considered as healthy. Factors from the social context were particularly relevant, namely, the food preferences or disease-related nutritional requirements of family members or social events (family reunions, social dinners, birthday parties) that were associated with traditions of rich food high in calories, fat and sugar. Access to a backyard garden can enable senior citizens to grow their own fruit and vegetables and was perceived as an opportunity to eat healthier food. Whereas the local shopping facilities were rated as satisfying even for particular food preferences, the restaurants in the (rural) neighbourhoods were criticised by interview partners for inducing them to eat oversized and unhealthy meals. On the macro level, the interview partners blamed the food industry and unsatisfying labelling policies for tricking them into buying food that may be detrimental to them (or to the environment or animal rights). Media coverage on healthy nutrition or consumer protection was reported to significantly impact on food choice and eating behaviour. 

Despite noticing that the environment affects their eating habits in an undesirable way, the interview partners did not seem to question those environmental factors but felt personally responsible for their eating behaviour. They dealt with undesired influences of the food environment predominantly by using strategies of adaptation and behaviour change on the individual level. Rather than challenging a social role (as a wife, as a host, as a guest) that is associated with eating or serving (unhealthy) meals that others prefer, they would make efforts to cook separate meals for themselves, or to go on a fast as compensation for over-indulging. Rather than confronting supermarket staff with the unsatisfying range of food in store, they go to ends to cycle to stores further off or grow their own vegetables. Especially with factors on the macro level (e.g., food industry), critical awareness was paired with the feeling of powerlessness.

### 4.2. Strengths and Limitations

The interview guide (Supplementary S1) allowed for thorough narrations and explanations of multiple factors influencing shopping and eating behaviour of the interview partners, thus reflecting the complexity of food environment as suggested by available frameworks. The sample represents a heterogeneous variety of an independent older population (including individuals of different gender, different socio-economic background and different marital status). In general, qualitative research approaches are not intended to be representative but rather to explore the range of concepts and perspectives based on the subjective views of specific groups of people. In qualitative research, the sample size is determined by theoretical saturation, i.e., the point when no new themes are brought up in the interviews [37]. This was reached after 35 interviews, so in terms of qualitative methodology, the sample size can be considered adequate. The selection of study participants, however, may have biased the results in certain ways. The answers of the interview partners were mainly determined by the fact that people were living in rural areas, e.g., concerning access to food retailers or ownership of a backyard to grow vegetables. Interviews with residents of larger cities may have yielded further contextual determinants of eating behaviour. According to a German study [38], it is more difficult to reach supermarkets by foot or public transport in rural areas as compared to urban areas. Accessibility of food retailers was described to be slightly worse in eastern Germany [38], so a study might have yielded slightly different or more pronounced results if performed in an Eastern German region. The food retailers are usually supermarket chains or discounter chains with a countrywide identical array of products and comparable architectural layout. As to the participants’ experiences in local restaurants, we would not expect different results in other parts of Germany. Whereas traditional cuisine varies across German regions, the different typical dishes have in common that they are hearty, fatty, rich in meat and often contain potatoes or potato products [39]. Therefore, the nutritional value of food to be obtained especially in many countryside inns can be expected to be similar across Germany [39]. In addition, the interview partners were recruited among the participants of a community-based project on healthy nutrition, so it can be assumed that the study population was especially interested in or committed to healthy nutrition. We conducted all interviews within the first two to three weeks of the project in order to have as little influence as possible on the participants’ answers. Still, this sample represents a selected sample of older people especially interested in the topic of nutrition. The positive aspect was that the interview partners had obviously reflected their diets before and were therefore able to give rather well thought-through, differentiated statements on this topic, which resulted in rich data material. As for those participants who were interviewed directly after the nutrition meetings, some aspects—which had been addressed or discussed in those meetings—may have still been in their minds and were referred to with a higher probability.

### 4.3. Comparison with Other Studies

To our knowledge, this is the first study exploring how older people in rural communities in Germany perceive and handle their food environment. We identified only a few studies that followed the complex ecological framework by Story et al. in a similar way. In a qualitative study, Deliens et al. [40] explored perceived determinants of eating behaviour of Belgian university students. Besides aspects that are characteristic of university live, e.g., student societies or exams, students described how the social environment (parental control, peer pressure), the physical (availability and accessibility of healthy foods and cooking supplies) and the macro environment (policy and legislation, media and advertising) influenced their eating behaviour. Watts et al. [41] used a similar approach, asking overweight adolescents which school- or community-related factors rendered healthful food choices easy or hard. Participants also mentioned several factors that can be assigned to the different levels, like peer influence (social level), food/beverage availability at school (physical level) or marketing influences (macro level) showing that the concept is also applicable to depict the perceived food environment of other subpopulations.

In our study, family members and changes in the household structure were perceived to have a substantial influence on eating habits. In a focus group study about US-American women’s perceptions about life events and diet in midlife, similar themes came up. The women reported positive and negative influences of household changes, family-related roles and influences of the health status of family members that led to changes in food choices [42]. Studies on widowhood emphasise that eating alone is a factor increasing nutritional risks in older adulthood [32,43]. This finding was not confirmed by the widowed or divorced older women in our study, who pointed out that being independent of their former partners’ food preferences improved the quality of their diets. This can be partly explained by the fact that the interview partners were a rather pro-active and mobile subpopulation, due to the recruitment strategy.

According to our interview results, there is no evidence for “food deserts” [44] in the rural regions where the study participants were recruited. Nevertheless, many factors of the physical environment that were found to influence the food choices among older adults in the rural western US [45] were in line with our findings. In the US-American study, older adults complained about insufficient public transportation to grocery stores, preferred shopping at the local farmers market due to better quality of fruit and vegetables and grew produce in their own vegetable garden. 

### 4.4. Relevance of the Study and Policy Implications

Although the senior citizens interviewed in our study were principally interested or determined to follow a diet they perceive as healthy, they all regularly experienced how their social, physical and societal environment renders this intention difficult, or even prevents healthy eating. This finding is reverberated in the statement of Nancy Milio, describing that “healthful lifestyles are not a matter of “free” choice, but rather the result of opportunities available to people, and... policy affects those opportunities” [46]. The health promotion approach as suggested by the World Health Organization takes up on this phenomenon and calls for policy endeavours that “make the healthy way the easy way” [6]. However, educational or behavioural approaches still predominate health promotion programs in the nutrition sector [47]. This also applies to interventions in communities, which often consist of advertising media campaigns or small group seminars [48]. Likewise, our study participants did not question their environment; neither saw they a need for it to change. Instead, they rather adapted to the environmental conditions, e.g., ate the meals the partner preferred in order to comply with their role as a caring wife, or they developed behavioural strategies to diminish negative environmental effects for themselves, e.g., by avoiding to go out for dinner completely because the restaurant cuisine was considered unhealthy or by walking to a far-off market to buy smaller packages of fruit or vegetables that were not in store in the closer supermarkets. This finding shows that health promotion programs should, in a first step, raise the awareness for environmental factors that are influencing eating habits not only among decision-makers, but also among citizens. People need to perceive their food environment as relevant and as potentially susceptible to change before they can engage in advocacy and activities to create healthier environments for themselves. Empowerment approaches seem to be promising. Empowerment is a core concept of the WHO vision of health promotion and can be defined as a multi-level process of gaining understanding and control over personal, social, economic, and political forces in order to take action to improve one’s life situations [49,50,51]. 

Beside empowering senior citizens to take action to improve their life situations, public health interventions in the field of healthy nutrition should address determinants that have been shown to be important in influencing eating habits. Community-based approaches could, for example, co-operate with local restaurants in order to help them add healthier items to the menu (Valdivia Espino et al., 2015). Media campaigns can point out that “festive” events do not necessarily need to be connected to heavy meals high in fat, sugar and calories, thus making “deviant” behaviour more socially acceptable. Supermarkets can be asked to develop special offers of fruit and vegetables that also appeal to single persons or small households, thus making this food affordable to senior citizens living alone [52].

By and large, the framework on food environment developed by Story was suitable to capture and categorise the determinants that were identified in the interviews. Nevertheless, we experienced overlaps between different levels in a number of occasions when analysing and categorising the interview statements. For example, a simple situation like eating out in a Bavarian restaurant could be assigned to the level of physical environment (lack of availability of healthier food in local restaurants); concurrently, it could also be seen as a factor on the social level because people ate out mainly because they were being invited to that restaurant for a social event. Likewise, a nutrition advice given by a local pharmacist or doctor is part of a social interaction between community member and health professional, but also refers to the level of the physical environment (availability of pharmacy and/or general practitioners in the neighbourhood). Therefore, the different levels that are central to the classification system appear to be artificial sometimes and cannot sufficiently display complex real-life situations.

## 5. Conclusions

The environmental influences on eating behaviour, as perceived by senior citizens, are manifold and partly inter-connected. In the studied population group, the social environment was considered the most dominant factor to be influencing eating behaviour. The group of older women has a particularly strong sense of social responsibility towards their husbands, their families and their friends, which determines their diets. They had difficulties in cooking and eating the way they thought healthy, because they felt trapped in traditional roles of wife, mother, guest or host, and for the most part, they simply adjusted to the nutritional preferences of their husbands, children or guests. Although the interviewed senior citizens experienced that their food environment impacted on their eating behaviour, they did not question or challenge the social or physical environment but accepted the limitations or took up an adaptive behaviour. Altogether, public health projects should raise the awareness of the multiple environmental influences on eating behaviour but also give people more self-confidence in order create healthier food environments for themselves.

## Figures and Tables

**Table 1:** Sociodemographic data of the participants (*n* = 35).

Sociodemographic Data of the Participants (*n* = 35)		Mean/*n* (%)
Age (in years)		71.2 ± 6.9
Gender	Female	29 (83%)
	Male	6 (17%)
Marital status	married/in relationship	20 (57%)
	divorced/widowed/single	15 (43%)

**Table 2:** The perceived food environment of older citizens in rural communities.

Perceived Environmental Factors Related to Nutrition			Which Are the Effects on Food Choice/Eating and Shopping Behaviour *(Positive + or Negative-, Perceived by Participants)*	How Do Participants Handle the Effects of the Perceived Food Environment *(If Mentioned)*
	Categories	Codes		
Social level	Preferences and needs of family members	Requirement of special diet due to Illness Food preferences or dislikes of spouses	Limitations in food variety (-)	Accepting higher effort in food preparation (e.g., cooking separate dishes)
	Reduction of household size	Quantitative and qualitative changes of purchasing and cooking habits	Cooking fewer helpingsAvoiding food which is disproportionately troublesome to prepare (e.g., baking wholemeal flour bread at home) (-) More freedom in food choice (+)	
	Expectations connected to certain roles (guest, host)	Association of social events (e.g., dinners, parties) with traditions of rich food	Cooking and/or eating high amounts of predominantly unhealthy food (-)	Controlling diet/weight, e.g., compensating high consumption of calories by fasting or eating only little during the day
	Influence of medical experts	Nutrition advice concerning healthy effects of special diets	Eating a special diet (e.g., low in cholesterol or salt) (+)	
Physical level	Access to an own backyard vegetable garden	High availability of home-grown, seasonal, (organic) food	Cooking and/or eating home-grown, seasonal, (organic) food (+)	
	Access to local food shopping facilities	Sufficient accessibility to supermarkets Limited accessibility due to insufficient public transportation (only in some communities)	Purchasing food in local shopping facilities (+) Limitations in shopping options (-)	Asking someone with a car to get a lift to the supermarket
	Available food in local shopping facilities	Poor availability of selected foods (e.g., fresh fish, whole meal flour) Annoying range, display and pricing schemes of produce and groceries	Not purchasing all the food as planned (-) Being enticed to buy sweets or low-cost big family portion sizes (-)	Organising shopping around the availability and accessibility of particular food items Shopping on farmer’s markets in the vicinity
	Cuisine of local restaurants	High density of traditional inns with plain fare and big portion sizes	Choosing unhealthy meals (e.g., energy-dense foods, meat) (-)	Avoiding eating out in restaurants Ordering special plates for seniors/take home leftovers
Macro-level	Food production/agriculture policies	Use of food additives and pesticides Lack of transparency, e.g., regarding meat procession	Uncertainty and doubts in shopping behaviour (-)	Changing food shopping behaviour (e.g., buying more regional products or less meat), hoping to put pressure on the food industry Cultivating produce in an own backyard vegetable garden
	Nutrition-related reports in mass media	Reports about healthy food	Trying new foods, e.g., stevia (+)	

## Supplementary Materials

The following are available online at https://www.mdpi.com/1660-4601/17/19/6940/s1, Supplementary S1: Interview guide.
