# Supplementary material for: How Older Citizens in Germany Perceive and Handle Their Food Environment—A Qualitative Exploratory Study
Source: Int J Environ Res Public Health. 2020 Sep 23;17(19):6940. doi: 10.3390/ijerph17196940 (PMC7579540; doi:10.3390/ijerph17196940)
Supplement: Supplementary file 1 [file ijerph-17-06940-s001.pdf]

Supplementary Material

Interview guide (english)

1) Please name factors you have experienced to influence your eating and food shopping habits
   or as a second version in case of no response:
   Please describe your typical eating behaviour on a workday and on weekends (prompts: buying food, preparing food, eating together/alone, eating out), and aspects that influence it
2) Please explain the relevance of family members with regard to food choice and eating behavior
3) Please refer to biographical events that have (recently) affected / changed food choice and diet
4) Do you have nutritional preferences? How have they developed?
5) Please point out actions that need to be taken to make healthy eating easier
6) Please explain what you do to make healthy eating easier for yourself or for others, e.g. family members.

Leitfaden (german)

1) Bitte nennen Sie Faktoren, die Ihre Ess- und Einkaufsgewohnheiten beeinflusst haben
   oder als zweite Version für den Fall, dass keine Antwort erfolgt:
   Erzählen Sie von ihrem typischen Ernährungsverhalten an einem normalen Wochentag und am Wochenende und gehen Sie dabei auf Faktoren ein, die dieses beeinflussen (*Erzählaufforderungen: Einkauf, Zubereitung, Esssituation: alleine, gemeinsam; Essensort: zu Hause, auswärts, unterwegs*).
2) Erklären Sie, welche Rolle Familienmitglieder in Bezug auf die Auswahl der Lebensmittel und das eigene Essverhalten spielen.
3) Nehmen Sie Bezug zu biographischen Ereignissen, die sich (vor kurzem) auf die Auswahl von Lebensmitteln und das eigene Essverhalten ausgewirkt haben.
4) Haben Sie Ernährungspräferenzen? Wie haben sich diese entwickelt?
5) Was müsste getan werden, um gesunde Ernährung einfacher zu machen?
6) Was tun Sie, um sich selbst oder ihren Angehörigen eine gesunde Ernährung zu erleichtern?
